# Strongly enhanced oxygen ion transport through samarium-doped CeO_2_ nanopillars in nanocomposite films

**DOI:** 10.1038/ncomms9588

**Published:** 2015-10-08

**Authors:** Sang Mo Yang, Shinbuhm Lee, Jie Jian, Wenrui Zhang, Ping Lu, Quanxi Jia, Haiyan Wang, Tae Won Noh, Sergei V. Kalinin, Judith L. MacManus-Driscoll

**Affiliations:** 1 Center for Nanophase Materials Sciences, Oak Ridge National Laboratory, Oak Ridge, Tennessee 37831, USA; 2 Center for Correlated Electron Systems, Institute for Basic Science (IBS), Seoul 151-742, Korea; 3 Department of Physics and Astronomy, Seoul National University, Seoul 151-742, Korea; 4 Department of Materials Science and Metallurgy, University of Cambridge, 27 Charles Babbage Road, Cambridge CB3 0FS, UK; 5 Department of Electrical and Computer Engineering, Texas A&M University, College Station, Texas 77843, USA; 6 Sandia National Laboratories, Albuquerque, New Mexico 87185, USA; 7 Center for Integrated Nanotechnologies, Los Alamos National Laboratory, Los Alamos, New Mexico 87545, USA

## Abstract

Enhancement of oxygen ion conductivity in oxides is important for low-temperature (<500 °C) operation of solid oxide fuel cells, sensors and other ionotronic devices. While huge ion conductivity has been demonstrated in planar heterostructure films, there has been considerable debate over the origin of the conductivity enhancement, in part because of the difficulties of probing buried ion transport channels. Here we create a practical geometry for device miniaturization, consisting of highly crystalline micrometre-thick vertical nanocolumns of Sm-doped CeO_2_ embedded in supporting matrices of SrTiO_3_. The ionic conductivity is higher by one order of magnitude than plain Sm-doped CeO_2_ films. By using scanning probe microscopy, we show that the fast ion-conducting channels are not exclusively restricted to the interface but also are localized at the Sm-doped CeO_2_ nanopillars. This work offers a pathway to realize spatially localized fast ion transport in oxides of micrometre thickness.

## Abstract (web summary)

Vertical nanocomposite films can exhibit a significant enhancement in oxygen ion conductivity, which is useful for oxide-based electrochemical devices such as fuel cells. Here, the authors directly probe this effect in high crystalline nanopillars using scanning probe microscopy.

## Graphical abstract

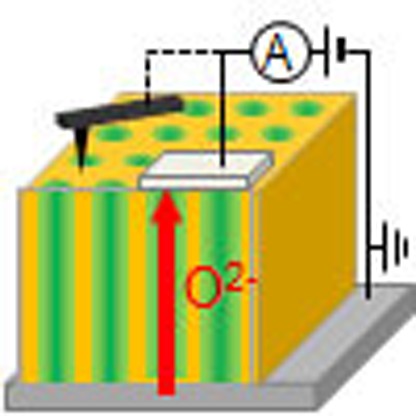

Fast oxygen ion transport at reduced temperatures is highly desired in many oxide-based electrochemical devices, including solid oxide fuel cells (SOFCs)^1^,^2^,^3^,^4^,^5^,^6^, oxygen separation membranes^7^, catalysts^8^ and memristors^9^,^10^,^11^,^12^. The current operation temperature of SOFCs is >800 °C because of limited oxygen ion conductivity in electrolytes and/or electrodes^1^,^2^,^3^,^4^,^5^,^6^. This results in high cell costs, long start-up and shut-down cycles and unacceptably fast performance degradation rates of SOFCs^3^,^5^. High oxygen ion conductivity is also directly linked to fast operation, high endurance and long retention in various ‘ionotronic (ionic + electronic)' devices, including memristors and ionically gated transistors^10^,^11^,^12^,^13^,^14^. Hence, enhancement of oxygen ion conductivity in oxides is a critical prerequisite for eventual near-room temperature operation of a wide range of ionotronic devices as well as optimization of their performance.

Tremendous efforts have been made to increase oxygen ion conductivity in oxides by designing artificial oxide heterostructures^2^,^4^,^5^,^6^. A notable example is ultrathin Y_2_O_3_-stabilized ZrO_2_ (YSZ) sandwiched by SrTiO_3_ (STO) layers, which were reported to exhibit eight orders of magnitude higher than the oxygen ion conductivity of bulk YSZ^2^. However, there has been much debate over the origin of such huge ionic conductivity, especially the nature of the charge carriers (that is, ionic or electronic)^15^,^16^,^17^,^18^,^19^,^20^. Shortly after the original report, Guo^15^ proposed that electronic p-type conductivity from the STO layer and the substrate might dominate the reported conductivity. García-Barriocanal *et al*.^16^ refuted this on the basis of the enormous difference between the ac and dc conductances. Cavallaro *et al*.^17^ grew the same heterostructures exhibiting similar enhancement of the conductance but interpreted the enhanced conductivity as having an electronic origin. More recently, however, De Souza and Ramaden^20^ analysed all of the reported data and showed that the conduction (in even STO) is exclusively ionic at temperatures below 540 K. In addition, Pennycook *et al*. reported density functional calculations and electron energy loss spectroscopy study, supporting that the nature of conduction is ionic^18^,^19^. These studies indicate that comprehensive and careful investigations are needed to clarify the underlying mechanism of ionic conductivity enhancement in oxide heterostructures and to answer basic questions, such as whether the conduction is ionic or electronic in nature and where the fast ion transport channels are located (for example, within individual layers, at their interfaces and/or even within the substrate).

In spite of the above issues, strain engineering and interface effects in oxide heterostructures have attracted considerable attention as a means to enhance oxygen ion conductivity. Recently, vertically aligned heterostructure films (referred to as nanoscaffold films here) have emerged as a very promising type of nanostructured systems for tuning the strain states of materials among other coupling effects^21^,^22^,^23^,^24^,^25^,^26^,^27^,^28^. They have great advantages compared with conventional lateral multilayer systems, such as high density of integration for devices and high strain tunability up to even micrometre thicknesses^21^,^22^,^23^,^24^,^25^,^26^,^27^,^28^. From a practical point of view, the vertical films are readily made by self-assembly, whereas the lateral multilayers require precise heterostructure engineering using sophisticated tools. Moreover, since the ion transport channels are not buried under the films, nanoscaffold films allow for direct probing of them with non-destructive tools, such as scanning probe microscopy (SPM)^29^,^30^,^31^, without the need for patterning, as illustrated in Supplementary Fig. 1. Such direct visualization of ionic conduction channels at the nanoscale combined with complementary structural and electrical measurements can provide further insight into the underlying mechanism of ion conductivity enhancement in oxide heterostructure films.

Here, oxygen ion transport in vertical heteroepitaxial micrometre-thick Sm-doped CeO_2_ (SDC)–STO films is explored using SPM. We apply electrochemical strain microscopy (ESM)^32^,^33^,^34^,^35^ and first-order reversal curve current–voltage (FORC-IV) methods^36^,^37^, allowing for local probing of ionic/electronic transport and associated electrochemical reactions at an ∼10-nm scale in nanoscaffold SDC–STO films. Macroscopic frequency- and temperature-dependent transport measurements, transmission electron microscopy (TEM) and X-ray diffraction measurements are also employed to give us a comprehensive picture of the nature of strongly enhanced nanoscale ionic conduction. The observed behaviours strongly indicate that the fast ionic conduction is not exclusively an interface phenomenon but rather resides in the whole volume of the high crystalline SDC nanopillars.

## Results

### High crystalline and epitaxial SDC nanopillars

**Figure 1:**
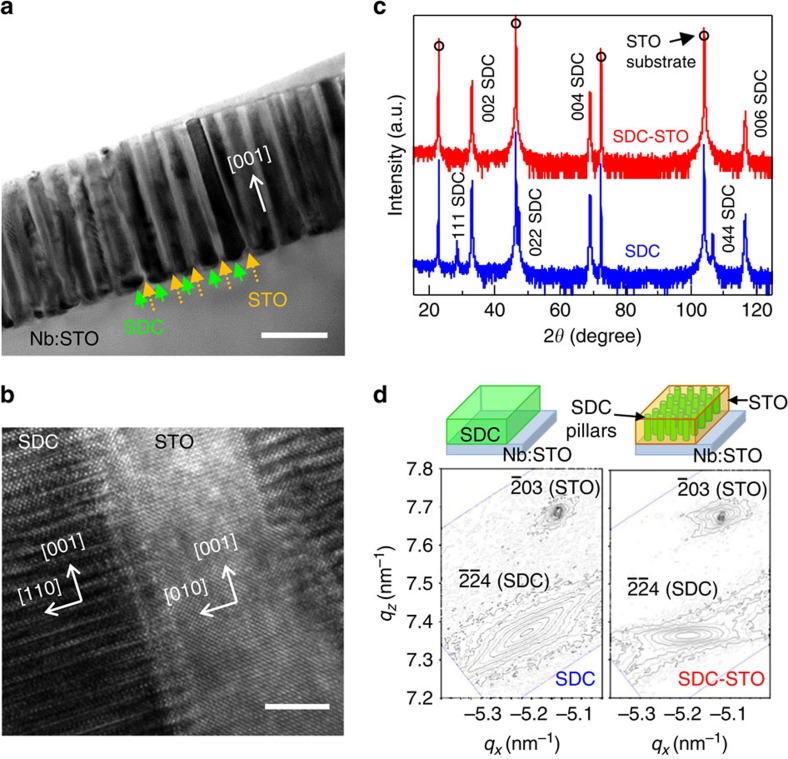
Crystal structure of the nanoscaffold SDC–STO film. (**a**) ‘Nano-comb'-like spontaneous phase ordering in cross-sectional view of the nanoscaffold SDC–STO film, as revealed by cross-sectional TEM image. Scale bar, 100 nm. (**b**) High-resolution TEM image of vertical SDC–STO interfaces in cross-sectional view. Scale bar, 10 nm. (**c**) Out-of-plane epitaxial relationship investigation in *θ*–2*θ* scan using X-ray diffraction. (**d**) Reciprocal space maps about the 
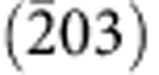
 STO substrate for a plain SDC film (left) and a SDC–STO nanoscaffold film (right).

Figure 1a shows a cross-sectional TEM image of the nanoscaffold SDC–STO film. The dark SDC nanocolumns (of ∼30-nm diameter) are uniformly distributed in the bright STO matrix. The nanopillars extend perpendicular to the Nb-doped STO (Nb:STO) substrate through the entire film thickness. Figure 1b shows a high-resolution TEM image of the vertical interfaces in cross-sectional view. The vertically aligned SDC–STO interfaces are very sharp. The SDC exhibits a fluorite structure (
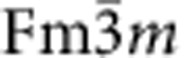
 in space group) in the 
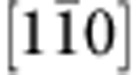
 direction, while the STO shows a perovskite structure 
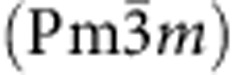
 in the [100] direction. An atomic-resolution high-angle annular dark field (HAADF) image of the SDC–STO interface, obtained using scanning TEM (STEM), is presented in Supplementary Fig. 2 (also see Supplementary Note 1).

The SDC nanocolumns are (001)-oriented on the Nb:STO substrate. The red line in Fig. 1c shows the X-ray diffraction pattern in a *θ−*2*θ* scan for an ∼1-μm-thick nanoscaffold SDC–STO film. In the 2*θ* range of 15°–125°, only SDC (00*l*) reflections are observed at 2*θ*=33°, 69° and 117° without any additional peaks indicative of no intermixing phases and solid solution phases. To compare the crystallinity of the nanoscaffold film, we grew a plain SDC film of the same thickness on a Nb:STO substrate. Unlike the nanoscaffold film, in the SDC film (the blue line in Fig. 1c), two minor peaks appeared at 28° and 47°, corresponding to SDC (111) and SDC (022) reflections, respectively. Hence, the thick SDC film does not grow epitaxially on the Nb:STO substrate. Since the {110} planes of STO match well to the {002} planes of the SDC, it is not surprising that pure (00*l*)-oriented SDC thick films are not easy to grow on (001) STO^38^. The full-width at half-maximum (FWHM) values for the (002) peaks for the plain SDC and nanoscaffold films were 0.8° and 0.5°, respectively. The smaller FWHM value in nanoscaffold films indicates little strain relaxation and hence low distribution of lattice parameters, indicating the higher crystallinity of the nanopillar SDC films compared to plain SDC films.

It is well known that nanoscaffold films are a powerful platform for preserving the strain state of a film with less thickness limitation, leading to achievement of high crystallinity^22^,^23^,^24^,^25^,^26^,^28^. This is because the strain in the nanoscaffold films relies on the vertical interphase coupling between the two materials that grow in the film, not the lattice mismatch between the film and substrate like in conventional lateral multilayer films. The lattice constants of cubic SDC and STO are 5.433 Å in JCPDS #750158 and 3.905 Å in JCPDS #730661, respectively. Thus, the SDC layers or nanopillars are grown on the Nb:STO substrate with a 45° in-plane rotation to minimize lattice mismatch, as demonstrated using a X-ray diffraction *φ*-scan (Supplementary Fig. 3). Figure 1d shows reciprocal space maps about the 
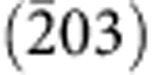
 STO substrate comparing the SDC film (left) and SDC–STO nanoscaffold film (right). In the ∼1-μm-thick SDC film, the substrate-induced strain is fully relaxed, showing a broad 
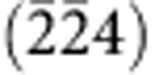
 SDC peak in the *q*_*z*_ axis (∼5.34–5.51 Å), that is, a wide spread of lattice parameters. The combination of the poor lattice match with the substrate and the strain relaxation in the films leads to the presence of the aforementioned polycrystalline phase with little strain in the film. On the other hand, the nanoscaffold SDC–STO film exhibits a very narrow 
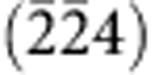
 SDC peaks in the *q*_*z*_ axis (5.42−5.44 Å), which is almost identical to the lattice constant of cubic SDC phase in the bulk. The results are consistent with the lower FWHM values of the (002) SDC peak measured for the nanocomposite film from above. Together, these results indicate that the SDC nanocolumn lattice is epitaxially controlled along the vertical direction by the surrounding STO matrix, an effect observed in other nanoscaffold film systems^22^,^23^,^24^,^25^,^28^.

### Ionic conductivity enhancement of nanoscaffold SDC–STO film

**Figure 2:**
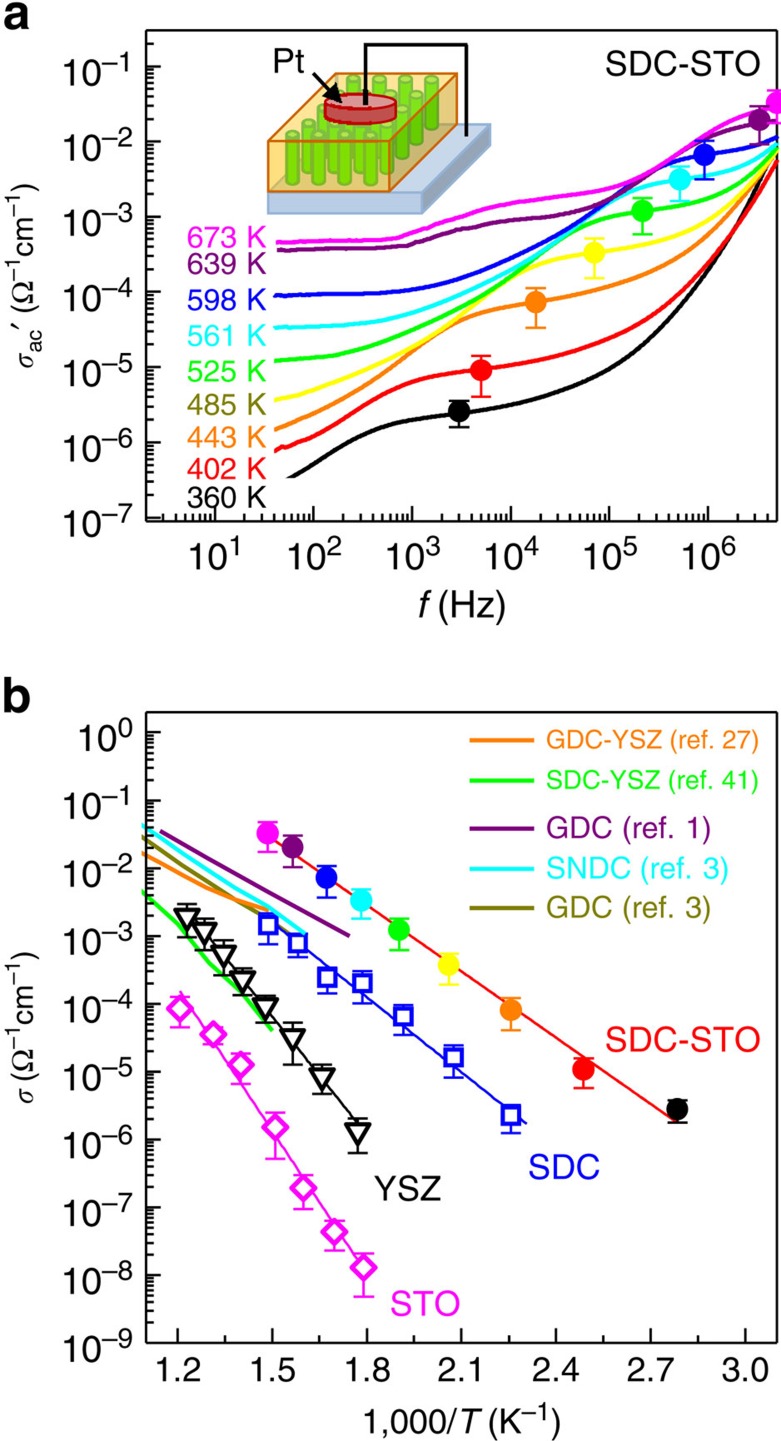
Transport properties of nanoscaffold SDC–STO films. (**a**) Frequency dependence of the real part of ac conductivity *σ*_ac_′ measured in the nanoscaffold SDC–STO film with variation of temperature. The ionic conductivity *σ* is defined from the plateaus of *σ*_ac_′, as indicated by solid circles. The error bars in the solid circles represent the possible spread in plateau positions. (**b**) Temperature dependence of *σ* for the nanoscaffold SDC–STO film. For comparison, we include those for the plain SDC, YSZ and STO films that we grew. The error bars of the *σ* values represent the small variation of the plateaus of *σ*_ac_′. We also include the *σ* values for GDC^1^,^3^, Sm_0.075_Nd_0.075_Ce_0.85_O_2−*δ*_(SNDC)^3^, GDC–YSZ nanocomposites^27^ and SDC–YSZ multilayers^42^ reported in the literature.

To investigate the macroscopic oxygen ion transport of the nanoscaffold films, we measured the frequency (*f*)-dependent ac conductivity *σ*_ac_, widely used for measuring ionic conductivity in disordered solids^2^,^39^. For the measurement, we deposited 150-nm-thick Pt top electrodes that are ion-blocking^40^,^41^. Figure 2a shows the double logarithmic plot of *f*-dependent real part of *σ*_ac_ (that is, *σ*_ac_′) of the nanoscaffold SDC–STO film in the *f*-range of 40–4 × 10^6 ^Hz. The *f*-dependent *σ*_ac_′ in the ionic conductors connected with ion-blocking electrodes exhibits the characteristic electrical response, as shown in Fig. 2a. Typically, *σ*_ac_′ decreases with decreasing *f* at high frequency because *σ*_ac_′ is governed by the short time ion dynamics (which is characterized by back-and-forth motion over the limited ranges) in the bulk^39^. At intermediate frequencies, *σ*_ac_′ is not a strong function of *f*, as displayed in the existence of plateau. It is well known that this distinct plateau is due to long-range ion transport, ‘diffusive' dynamics, providing the dc conductivity of ions^2^,^39^. The plateaus of *σ*_ac_′ shift up with an increase in temperature, as represented by the solid circles in Fig. 2a. At low frequency, *σ*_ac_′ decreases again with decreasing *f* since oxygen ions are blocked by the Pt top electrodes and accumulate there, leading to the formation of space-charge layers and rapid voltage drop in such layers, the so-called ‘electrode polarization' effect (Supplementary Fig. 4 and Supplementary Note 2)^39^. These characteristic *f* dependences of *σ*_ac_′ suggest that the nature of conduction is mainly ionic in nanoscaffold films. To prove the ionic nature of conduction in the nanoscaffold SDC–STO films, we measured the conductivity of the samples by means of a dc technique^2^. As can be seen in Supplementary Fig. 5, the measured dc conductivity (open circles) is approximately two orders of magnitude lower than the values obtained from *σ*_ac_ measurements (solid circles) in the entire temperature measurement range. This result shows that the electronic contribution to the ac impedance measurements can be considered to be negligible and, thus, that the measured ac transport is dominated by oxygen ions, not by electrons or holes (also see Supplementary Note 3).

We found that oxygen ion conductivity of nanoscaffold SDC–STO films is higher than those of other plain SDC, STO and YSZ films for all of the temperature range measured (Fig. 2b). As noted before, the plateaus of *σ*_ac_′, represented by solid circles in Fig. 2a, allow us to determine the values of oxygen dc ion conductivity *σ* at each temperature^2^,^39^. The *σ* value of the nanoscaffold film reaches as high as 3 × 10^−2 ^Ω^−1 ^cm^−1^ at 400 °C (the cross-sectional area for *σ* values was calculated based on the SPM results that the ion-conducting channels are localized in the whole area of SDC nanopillars, as discussed below). For comparison, we grew individual SDC, YSZ and STO films (see Methods) and measured their *σ* values by means of the same procedure as the nanoscaffold film. As shown in the guidelines, the temperature dependences of *σ* follow the well-known Arrhenius behaviour in all samples. The estimated *σ* values of SDC, YSZ and STO were ∼10^−3^, 10^−4^ and 10^−6 ^Ω^−1 ^cm^−1^ at 400 °C, respectively. Namely, the oxygen ion conductivity in the nanoscaffold film is much higher than that in other comparison films by one to four orders of magnitude, indicative of considerable enhancement of ionic conductivity in the nanoscaffold films. In addition, our nanoscaffold SDC–STO films have higher ionic conductivity than other CeO_2_-based ionic conductors and heterostructures reported in the literature (solid lines in Fig. 2b), such as Gd_0.1_Ce_0.9_O_*x*_ (GDC)^1^,^3^, Sm_0.075_Nd_0.075_Ce_0.85_O_2−*δ*_ (ref. 3), SDC–YSZ multilayers^42^ and GDC–YSZ nanocomposites^27^. Note that the values of activation energy for oxygen migration in the nanoscaffold SDC–STO film and the plain SDC film are 0.65±0.02 and 0.70±0.04 eV, respectively. Although the value of the SDC–STO film is slightly smaller than that of the plain SDC film, they were not distinguishably different within the error bars. This indicates that the reduction of activation energy is not the origin of ionic conductivity enhancement in our SDC–STO films.

### Nanoscale mapping of electrochemical oxygen redox process

**Figure 3:**
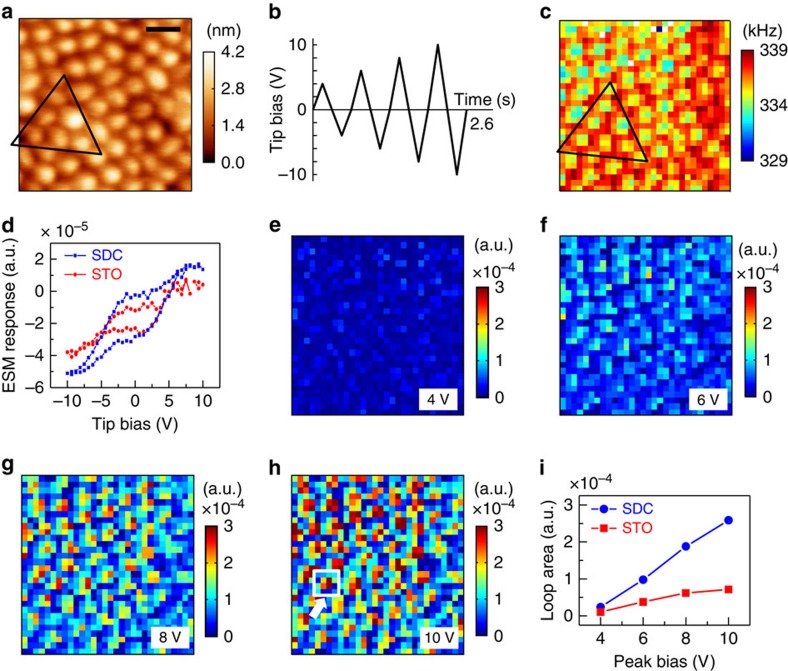
Nanoscale mapping of electrochemical oxygen redox process at room temperature. (**a**) Topographic image of the nanoscaffold SDC–STO film surface. The bright circular regions of ∼30 nm diameter are the SDC columns intersecting the film surface. The image size is 300 × 300 nm^2^. Scale bar, 60 nm. (**b**) The bias waveform used for ESM measurement. (**c**) Spatial map of the ESM resonance frequency analysed using a simple harmonic oscillator model fitting, measured at the dc bias of −7 V. The open triangles in **a**,**c** show that the probing regions are identical. (**d**) ESM hysteresis loops with the maximum voltage of 10 V, averaged over one particular SDC column and its surrounding STO region, represented by the open box in **h**. (**e**–**h**) Spatial maps of ESM hysteresis loop area as a function of the peak bias. (**i**) ESM loop areas as a function of the peak bias averaged over the 30 points of the SDC and STO regions.

To investigate the primary origin of oxygen ion conductivity enhancement in the nanoscaffold SDC–STO films, the local bulk ionic transport and associated interfacial oxygen reduction/evolution reactions (ORR/OER) were probed directly at the nanoscale. We first performed FORC-ESM measurements at room temperature, enabling nanoscale mapping of the electrochemically active regions^34^,^43^. Figure 3a shows a topographic image (300 × 300 nm^2^) of a ∼230-nm-thick nanoscaffold film top surface. Bright circles (∼30-nm diameter, independently revealed with TEM work) and dark surrounding region indicate SDC nanopillars and STO matrix, respectively. In the FORC-ESM measurements, the amplitude of a triangular wave was linearly increased until the maximum value of 10 V was reached (Fig. 3b). The ESM responses were recorded over a grid of 30 × 30 points (thus, the size of one point is 10 × 10 nm^2^). Further experimental details are described in Methods. Figure 3c shows the spatial map of contact resonance frequency *f*_cr_, which is one of the key pieces of information obtained from the measurement. The *f*_cr_ value in the STO matrix is relatively high and uniform, whereas that in SDC nanocolumns is low and inhomogeneous. Since *f*_cr_ is very sensitive to the changes in mechanical properties of the tip–surface junction, the variation of *f*_cr_ within SDC nanocolumns is attributed to chemical inhomogeneity of the SDC phase (Ce^3+^, Ce^4+^ and Sm^3+^) because of the doping of them by Sm and surface hydroxylation^44^. In contrast, STO is expected not to have significant cation inhomogeneity, resulting in nearly uniform *f*_cr_ values in the STO region. Moreover, we found clear radial direction-dependent *f*_cr_ values in the SDC nanocolumns (see Supplementary Fig. 6); the pillar core has a smaller *f*_cr_ value than its outskirts. Such a *f*_cr_ gradient along the radial direction implies that the concentration of oxygen vacancies varies along that direction.

We observed that SDC nanopillars have higher ORR/OER rates than the STO matrix. Figure 3d shows typical ESM hysteresis loops with the maximum voltage of 10 V, averaged over one particular SDC column (corresponding to the 4 points) and its surrounding STO region (12 points), as indicated by the open box in Fig. 3h. The ESM hysteresis loops come from the molar volume change induced by tip-bias-activated interfacial ORR/OER process and subsequent oxygen ion diffusion or electromigration^34^. The hysteresis loop measured in the SDC nanocolumn has a larger area and height than that in the STO region. The area and height of the hysteresis loops are directly linked to the changes in oxygen ion concentration during the voltage cycle based on the empirical Vegard's law^34^,^45^. Figure 3e–h shows the evolution of spatial maps of the ESM hysteresis loop area with an increase in the peak bias, that is, the maximum voltage of each triangular pulse. The ESM response loop opened up as the bias voltage increased. It is obvious that SDC columns show a much larger hysteresis loop evolution than the STO region. Figure 3i plots the ESM loop area as a function of the peak bias averaged over the 30 points of the SDC and STO regions. The slope of the curve relative to the SDC region is noticeably higher, but that of the STO region is smaller. Therefore, the ORR/OER process is more active in the SDC nanocolumns. It is worth noting that the onset voltages for the ORR/OER process were similar irrespective of SDC and STO regions (Supplementary Fig. 7). This suggests that thermodynamic potential for the activation of ORR/OER is nearly the same in the probed SDC and STO regions. Thus, the difference of electrochemical activity would be due to the amount of oxygen ion concentration, not the reduction of activation energy.

### Nanoscale visualization of ion-conducting channels

**Figure 4:**
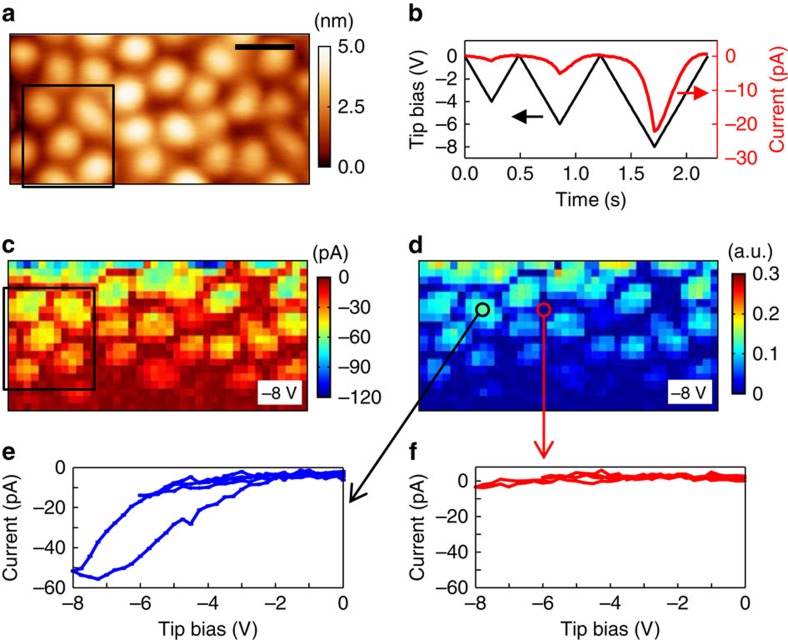
Spatially resolved mapping of ion-conducting channels. (**a**) Topographic image of the nanoscaffold SDC–STO film. The image size is 250 × 125 nm^2^. Scale bar, 50 nm. (**b**) The bias waveform used for the FORC-IV measurement and time-dependent current response averaged over the 40 × 20 points. Spatial maps of (**c**) current response and (**d**) relative FORC-IV loop area measured at −8 V. The boxed regions in **a**,**c** show that the probing regions are identical. Current versus tip bias curves at (**e**) the SDC nanopillars core and (**f**) the STO region.

To visualize directly nanoscale oxygen ion-conducting channels in the nanoscaffold films, we proceeded to perform FORC-IV measurements (see Methods)^36^,^37^. Since the FORC-IV method detects the electrical current flowing between the tip and the bottom electrode (here Nb:STO), it provides direct information of ionic and/or electronic conduction. We found that a high bias (≥27 V) was required to detect noticeable currents at room temperature (Supplementary Fig. 8). To reduce the magnitude of applied bias and to enhance the signal-to-noise ratio, we performed the measurements at a relatively high temperature of ∼110 °C. Figure 4a shows a topographic image (250 × 125 nm^2^) of the nanoscaffold film top surface. Owing to asymmetric current behaviour depending on the polarity of dc bias, that is, high (low) current at negative (positive) bias (Supplementary Fig. 8), we carried out the negative bias FORC-IV measurements to get high signal-to-noise ratio. We used the bias waveform illustrated in Fig. 4b. Note that the application of negative bias to the tip activates ORR at the tip–surface junction. The average *I–V* response averaged over a grid of 40 × 20 points (thus, the size of one point is 6.25 × 6.25 nm^2^) showed an initial non-hysteretic curve that gradually transformed into a hysteretic one above −6 V (Supplementary Fig. 9).

Contrary to the average behaviour, the local current exhibits high spatial variability. Figure 4c shows the spatial map of current response at tip bias of −8 V. Highly conductive SDC columns and nearly insulating STO regions are present (also see Supplementary Fig. 10). It is worth noting that the current signal became degraded during the measurement (the measurement sequence was from top left to bottom right) presumably due to the degradation of tip metal coating. However, it did not affect the main result that only the SDC nanopillars are conductive. The relative FORC-IV loop area enables us to estimate the electrochemical activity; a larger relative loop area indicates a higher electrochemical activity^36^,^37^. As shown in Fig. 4d, the SDC columns show high electrochemical activity, consistent with the previous FORC-ESM results. In particular, the core of SDC column has a larger relative loop area compared with the outskirts of column. This trend was also observed in the current map. Considering the finding of a *f*_cr_ gradient along the radial direction, which was observed in the FORC-ESM measurement, the results indicated that the SDC column core has more mobile oxygen vacancies. Figure 4e,f shows the individual *I*–*V* curves at the pixels corresponding to the core of SDC nanocolumn and STO region, respectively. The core of the SDC column exhibits high current and clear loop opening, whereas the STO region shows nearly zero current. Therefore, it can be concluded that oxygen anionic current is well localized to the SDC nanocolumns in the nanoscaffold film. In addition, it can be confirmed again that the electronic contribution of the STO component of the film to the enhancement of ionic conductivity is negligible, since the STO matrix does not exhibit any conduction (either ionic or electronic).

## Discussion

Spatially resolved mapping of oxygen ionic conduction at the nanoscale reveals that only the SDC nanopillars have high interfacial ORR/OER rates as well as significant bulk oxygen ion conductivity. Especially, the highest ion conductivity is observed at the column centre, rather than at the SDC–STO interface. This contrasts with the common consensus that the interfaces in planar oxide heterostructure films act as the fastest ion transport pathways^2^. It should be noted that, in the vertical BiFeO_3_–CoFe_2_O_4_ nanocomposite systems^30^,^31^ and the SrTiO_3_–Sm_2_O_3_ nanocomposite system^29^ that we have studied earlier for memristor effects, the enhanced conduction was observed at the interface regions. However, the materials in those films were not good oxygen ionic conductors. Instead, oxygen vacancies and hence oxygen conduction arose from interface strain and structural incompatibility. In this study, on the other hand, the SDC phase is already a very good ionic conductor with a high concentration of oxygen vacancies, and it can be expected that its ‘bulk' behaviour dominates the conduction. In addition, the direct visualization of ionic conduction rules out the electronic contribution of the surrounding STO matrix to ionic conductivity enhancement, which is another debatable issue in planar oxide heterostructure films. Therefore, the precise determination of the location of the dominant ion-conducting region is essential for the analysis of ionic conductivity.

Although the interfaces are not the fastest ion-conducting channels in our nanoscaffold films, the interfacial coupling by the surrounding STO matrix plays a critical role in the enhancement of oxygen ion conductivity. The primary origin of oxygen ion conductivity enhancement in nanoscaffold SDC–STO films is the high crystallinity of SDC nanopillars through the whole micrometre-thick film, which is controlled by the interphase epitaxial coupling to the STO matrix. We recall that for the nanoscaffold films, there is a narrower (002) SDC X-ray peak and much narrower RSM peak width of 
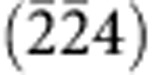
 peak along *q*_*z*_ (Fig. 1d). Hence, the interplanar spacing along the length of the nanorods is very uniform in the nanoscaffold film. In conventional lateral plain films, the substrate-induced elastic strain is typically limited to only tens of nanometre, which is around the dimensions of the SDC nanocolumns here. Such strain relaxation reduces crystallinity of the films and generates a variety of nanoscale imperfections, such as structural misfit or edge dislocations^46^ and grain boundaries^6^,^42^,^47^,^48^,^49^,^50^,^51^ (Supplementary Fig. 11), which can perturb the ionic conduction path. Recently, Sun *et al*.^46^ showed that edge dislocations slow down oxygen ion diffusion in doped CeO_2_ by segregation of charge defects. In addition, it has been reported that the grain boundaries in CeO_2_ have lower ionic conductivity than the bulk both experimentally^42^,^47^,^48^,^49^,^50^ and theoretically^51^. On the other hand, crystal perfection of SDC nanopillars achieved by the interfacial coupling to the STO matrix can form ‘oxygen migration highways' along the *c*-axis direction by suppressing such inhomogeneities.

Finally, since the diameters of the SDC nanopillars are only ∼30 nm, we should consider the effect of space-charge regions at the SDC–STO interfaces and the possibility that they could overlap and therefore influence the oxygen ion conductivity. When two different phases meet, a highly ionic conductive path can be created along the interfaces as a result of ion redistribution in a space-charge region^52^,^53^,^54^. Especially, as demonstrated in the lateral CaF_2_/BaF_2_ heterostructure films^53^, if the spacing between different layers is reduced further, the space-charge regions at the interfaces overlap, enabling further increase in ion conductivity because of mesoscopic size effects. However, as directly probed in the SPM measurements, in our nanoscaffold films the fastest ion transport channels are located at the centres of SDC nanopillars, away from the interfaces. In addition, it is expected that the widths of the space-charge regions formed at the interface are very narrow (below 1 nm) in the heavily doped ionic conductors such as YSZ and SDC^55^. Thus, it would be not possible for the space-charge regions to overlap inside the SDC nanopillars and have the maximum ion conductivity at the centres of pillars. It is worth noting that, as pointed out in ref. 55, the additional accumulation of charge carriers at the space-charge regions in the heterostructures made of heavily doped oxides cannot significantly affect the total conductivity compared with large bulk defect concentrations because the width of the space-charge regions will be too small.

In conclusion, we have explored the oxygen ion transport in micrometre-thick vertical nanocomposite SDC–STO films. The macroscopic ionic conductivity in nanoscaffold films was higher than those in plain SDC, YSZ and STO thick films by up to four orders of magnitude. We have investigated interfacial ORR/OER process as well as bulk oxygen ion transport using SPM techniques because of the unique nanoscaffold geometry studied here where conduction channels can readily be probed, which is not the case for buried interfaces in standard planar films. Spatially resolved mapping of oxygen ion transport at the nanoscale revealed that only the SDC nanopillars have high oxygen ion conductivity, while the surrounding STO matrix showed negligible conduction. On the basis of these SPM results combined with complementary macroscopic measurement results, the high crystallinity of SDC nanopillars comparable to a single SDC phase is the primary origin of oxygen ion conductivity enhancement in nanoscaffold films. Note that such high crystalline quality of epitaxially grown SDC nanopillars without crystalline imperfections was achieved by interfacial strain coupling of the SDC to the surrounding STO matrix through the whole micrometre-thick film.

This work highlights that the crystalline quality of bulk ionic conductors is very important for ionic conductivity enhancement in oxide heterostructures, a fact that is often overlooked. In particular, the conduction in nearly single-crystalline bulk phase materials can be more dominant in oxide heterostructures composed of heavily doped ionic conductors. In addition, direct spatially resolved mapping of oxygen ion conduction at the nanoscale is needed to verify the underlying mechanism of ionic conductivity enhancement. The vertical nanocomposite structures studied here provide unique structures that allow for easy probing of interface and bulk regions. They also represent a simple, self-assembled system for realizing micrometre-thick fast ionic conduction channels and are widely applicable for clean energy, multifunctional ionotronic and novel information devices.

## Methods

### Film growth

The SDC–STO nanoscaffold films were grown to 0.5% Nb:STO (001) single crystal by a simple one-step process of pulsed laser deposition. We ablated a polycrystalline target containing SDC (20% wt. SDC) and STO (50:50 molar ratio) by a KrF excimer laser (248-nm wavelength) with a laser fluence of 1.5 J cm^−2^. During the deposition, we heated the substrate at 825 °C and flowed 200 mbar oxygen. The samples were post annealed at 700 °C for 1 h under 900 bar oxygen to assure proper oxygen stoichiometry and to minimize the superfluous oxygen vacancies inside films. We selected 0.5% Nb:STO (001) single crystal as the substrate since it has been considered as a SOFC anode material because of redox stability and electrochemical properties^40^,^56^. The Nb:STO single crystal also acts as a bottom electrode for ESM and FORC-IV measurements. We also fabricated single SDC, STO and YSZ films under the same deposition procedure using SDC, STO and YSZ (8% wt. YSZ) polycrystalline targets, respectively. We deposited Pt top electrodes by dc magnetron sputtering for transport measurements.

### Characterization

The microstructural characterization has been conducted using TEM (FEI Tecnai G2 F20) as well as aberration-corrected STEM (FEI Titan G2 80–200), both operated at 200 kV. STEM HAADF images were taken using an annular detector with a collection range of 60–160 mrad. Cross-sectional samples for TEM characterization were prepared by a standard grinding and thinning and a final ion-milling (Gatan PIPS 691 precision ion polishing system). The X-ray diffraction was carried out with a Panalytical Empyrean high-resolution X-ray diffractometer using Cu-*Kα* radiation (*λ*=1.5405 Å). To measure transport characteristics with temperature variation from 20 to 550 °C, we used a probe station equipped with a hot plate. The ac and dc measurements were performed by an HP 4294A Precision Impedance Analyser and Keithley 2,440 sourcemeter, respectively. For all transport measurements, we grounded the Nb:STO substrate and applied the voltage to the Pt electrodes.

### SPM measurements

ESM and FORC-IV measurements were performed using a commercial atomic force microscope (Cypher, Asylum Research) interfaced with National Instrument cards controlled by the Labview and Matlab software. The signal was applied to the cantilever with a conductive Pt/Cr coating (Budget Sensors, Multi75E-G). Image and data processing were performed using WSxM (ref. 57) and custom-written Matlab codes, respectively. In ESM, the conductive tip confines an electric field in a nanoscale volume of material, activating the local ORR/OER processes and subsequent oxygen ion (or vacancies) transport. Then, the tip detects the associated change in molar volume, that is, electrochemical strain at a few picometre levels^34^. To enhance the signal-to-noise ratio and mitigate the topographic crosstalk, we used the band-excitation ESM method^58^. Briefly, an electric bias with a band of frequencies around the contact resonance of the cantilever was applied, and the response was measured and Fourier-transformed simultaneously to yield the total ESM response. It was then analysed using a simple harmonic oscillator model fitting to extract signals for amplitude, phase, resonance frequency and quality factor. The FORC-IV measurement is based on the local current measurement and employs a bias waveform consisting of a sequence of triangular pulses with increasing amplitude^36^,^37^. The current through the sample was measured using a commercial current amplifier (Femto, DLPCA-200). For high-temperature measurements, we used environmental scanner (Asylum Research).

## Additional information

**How to cite this article:** Yang, S. M. *et al*. Strongly enhanced oxygen ion transport through samarium-doped CeO_2_ nanopillars in nanocomposite films. *Nat. Commun.* 6:8588 doi: 10.1038/ncomms9588 (2015).

## Supplementary Material

Supplementary Information Supplementary Figure 1-11, Supplementary Note 1-3 and Supplementary References.
