# Supplementary material for: Strongly enhanced oxygen ion transport through samarium-doped CeO2 nanopillars in nanocomposite films
Source: Nat Commun. 2015 Oct 8;6:8588. doi: 10.1038/ncomms9588 (PMC4633963; doi:10.1038/ncomms9588)
Supplement: Supplementary Information — Supplementary Figure 1-11, Supplementary Note 1-3 and Supplementary References. [file ncomms9588-s1.pdf]

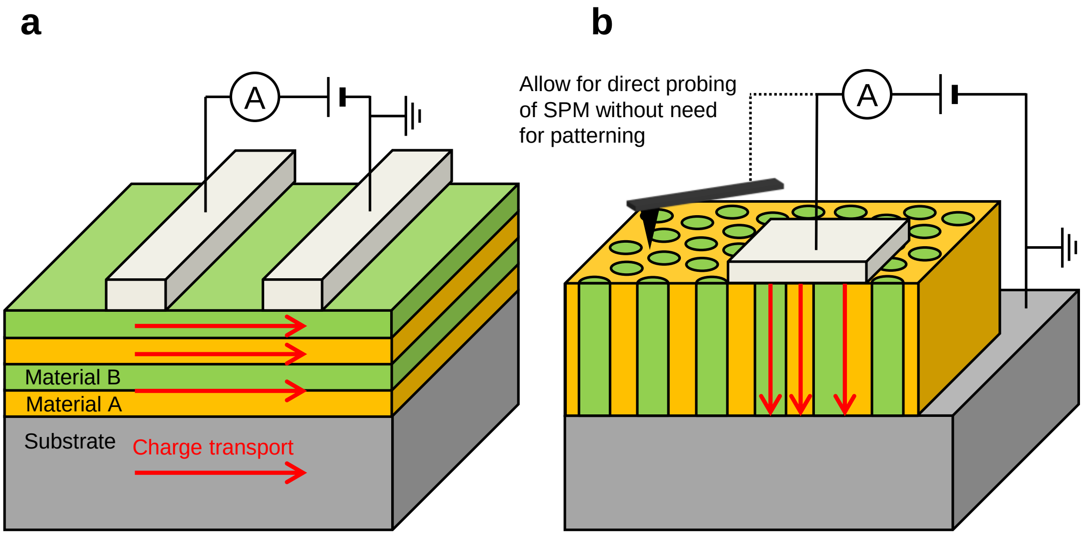

Supplementary Figure 1: Schematic illustrations of possible charge transports in multilayers and nanoscaffold films. (**a**) A conventional lateral multilayer film and (**b**) a vertically aligned nanoscaffold film. Nanoscaffold films allow for direct probing of the charge transport channels with non-destructive tools, such as scanning probe microscopy (SPM), without the need for patterning.

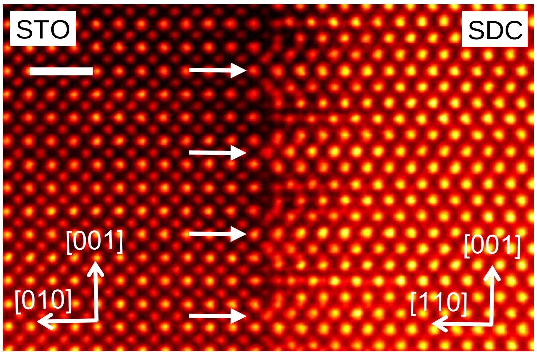

Supplementary Figure 2: Atomic-resolution HAADF image at the SDC/STO interfaces by STEM. Image take along the [100] STO and [1$\bar{1}$0] SDC directions. Scale bar, 1 nm. The white arrows indicate the periodic coherency between the lattices of SDC and STO. A detailed description of the strain state and oxygen sublattice at the SDC/STO interface is described in the Supplementary Note 1.

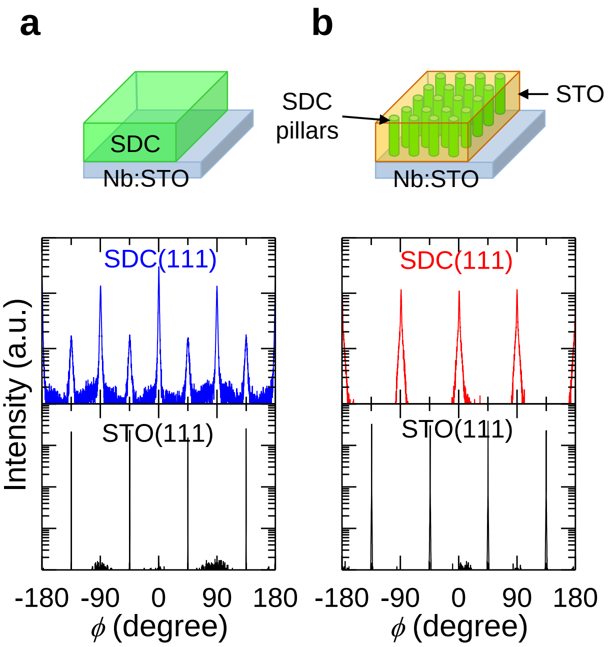

Supplementary Figure 3: In-plane epitaxial relationship investigation by x-ray phi scans. The top and bottom panels show 360° phi scans of the SDC (111) film or nanopillars and STO (111) substrate reflections, respectively. (**a**) For the plain SDC film, four SDC (111) reflections are shifted from $SrTiO_3$ (111) by 45°, indicative of SDC[110]‖STO[100]. However, additional four reflections also exist on $SrTiO_3$ (111), suggesting that the epitaxial growth is also possible with {110} planes of the STO matching the {002} planes of the SDC. (**b**) For the nanoscaffold SDC-STO film, the film and substrate peaks are separated by 45°, revealing the in-plane relationship of SDC[110]‖STO[100].

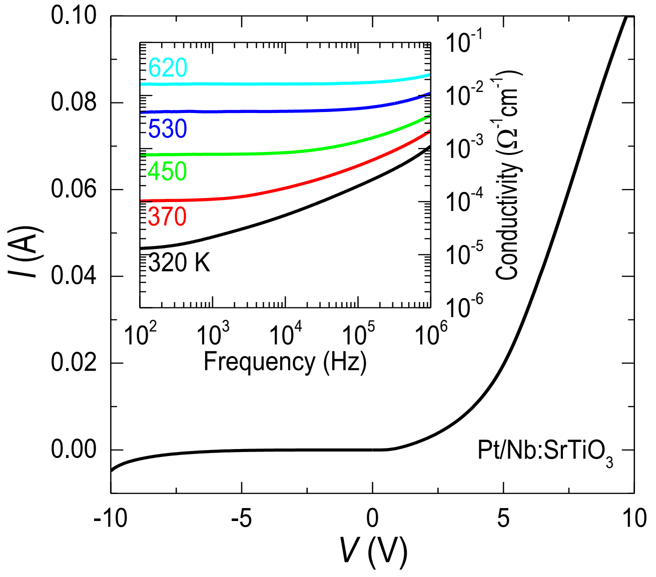

Supplementary Figure 4: Current-voltage curve and frequency-dependent real part of the ac conductivity in a Pt/Nb:$SrTiO_3$ system. Forward rectifying current−voltage curves in the Pt/Nb:$SrTiO_3$ system indicate the formation of the Schottky barrier at the interface. The inset shows the frequency-dependence of the real part of ac conductivity (σ_{ac}'). A drop of ac conductivity is not observed at low frequency, which is different from the SDC-STO nanoscaffold films (Please see Fig 2a in the main text). A detailed description of the f-dependent σ_{ac}' in the Schottky system is described in the Supplementary Note 2.

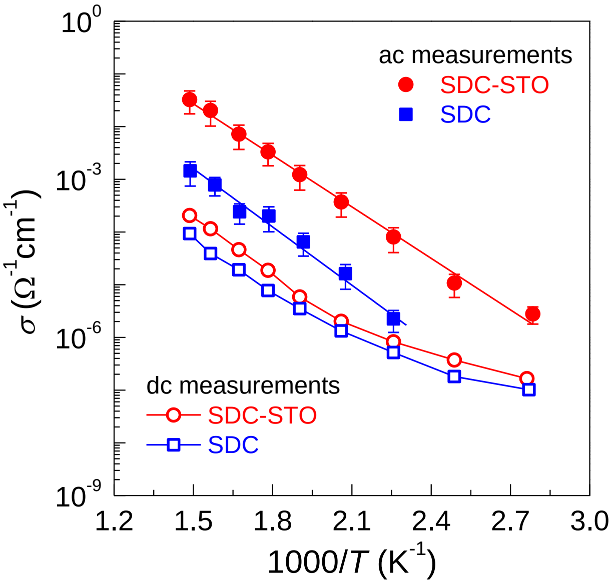

Supplementary Figure 5: Comparison of conductivities measured by dc and ac techniques. Arrhenius plot of the dc conductivity of the SDC-STO nanoscaffold film obtained by dc (open circles) and ac (solid circles) measurements. For comparison, the dc conductivity of the plain SDC film obtained by dc (open squares) and ac (solid squares) measurements are also included. The error bars of the dc conductivities from ac measurements (solid symbols) represent the small variations of the plateaus since the plateau regions are not very flat (see Fig. 2a of the main text). A detailed description of the comparison of dc conductivity is described in the Supplementary Note 3.

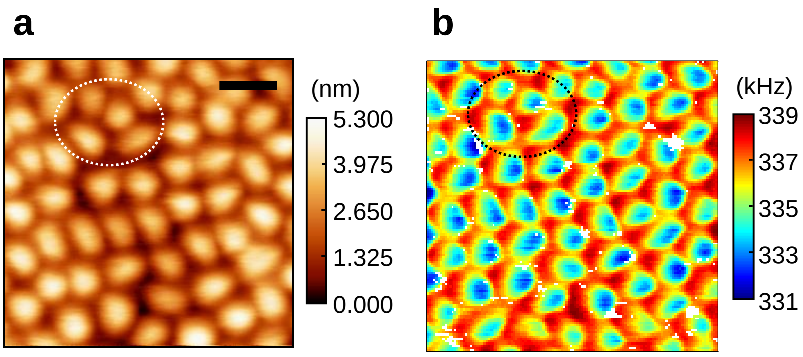

Supplementary Figure 6: Band-excitation (BE) electrochemical strain microscopy (ESM) imaging. (**a**) Topographic image of nanoscaffold SDC-STO film surface. The bright circular regions of 30-nm diameter and the dark surrounding region indicate the SDC nanopillars and the STO matrix, respectively. The image size is 300 × 300 nm^2. Scale bar, 60 nm. (**b**) Spatial maps of the BE-ESM contact resonance frequency (f_{cr}) analysed using a simple harmonic oscillator model fitting. The open dotted ellipses in (**a**) and (**b**) represent that the probing region are identical. The STO matrix has a uniform f_{cr} value, while the SDC nanocolumns have the radial direction-dependent f_{cr} values; the column core has a smaller f_{cr} value than its outskirts. This is presumably due to the chemical inhomogeneity of SDC due to doping of Sm. For detailed discussion, please see the main text.

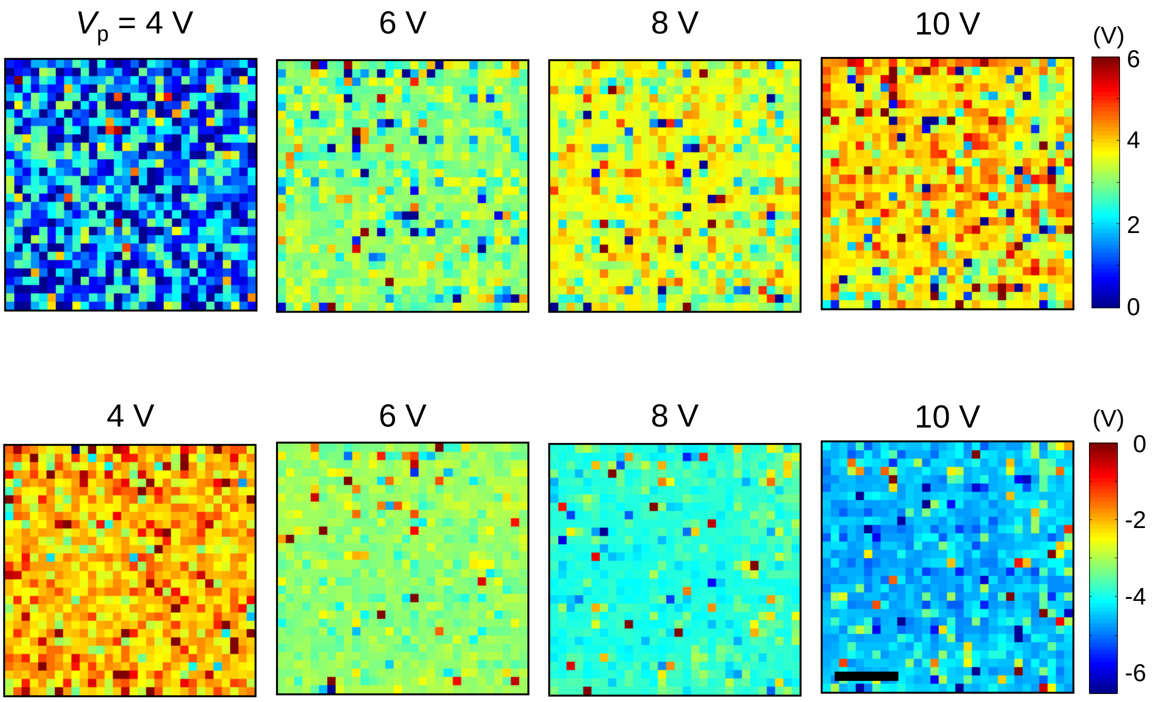

Supplementary Figure 7: Onset voltage for oxygen reduction/evolution reactions (ORR/OER). Spatial maps of onset voltages for ORR/OER processes occurring at the tip-surface junction as a function of the peak bias V_p, that is, the maximum voltage of each triangular pulse. The image size is 300 × 300 nm^2. Scale bar, 60 nm. Overall, they are approximately homogeneous, i.e., we cannot see any significant difference depending on the position. This indicates that the thermodynamic potential for the activation of ORR/OER is similar in the probed SDC and STO regions.

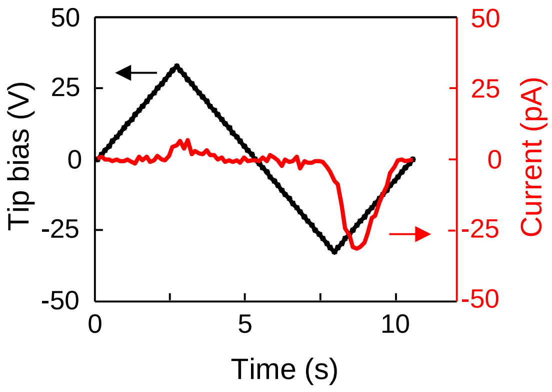

Supplementary Figure 8: Bipolar *I-V* measurement at room temperature. The current response as a function of time and the bias waveform used for the measurement. The peak biases (that is, the maximum voltage of each triangular pulse) are ± 30 V.

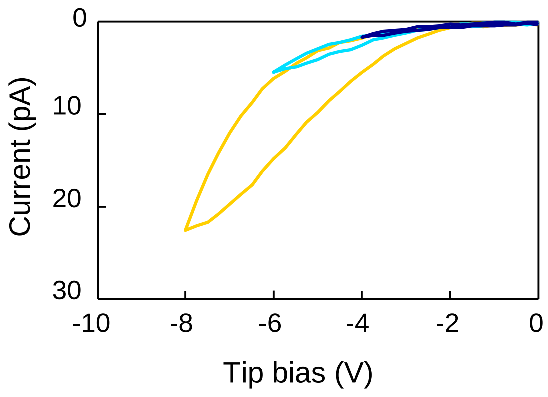

Supplementary Figure 9: Negative bias FORC-IV measurement at a relatively high temperature of ~ 110 °C. The current response is measured as a function of tip bias. Initial non-hysteretic curve with negligible current is gradually transformed into a hysteretic one above -6 V.

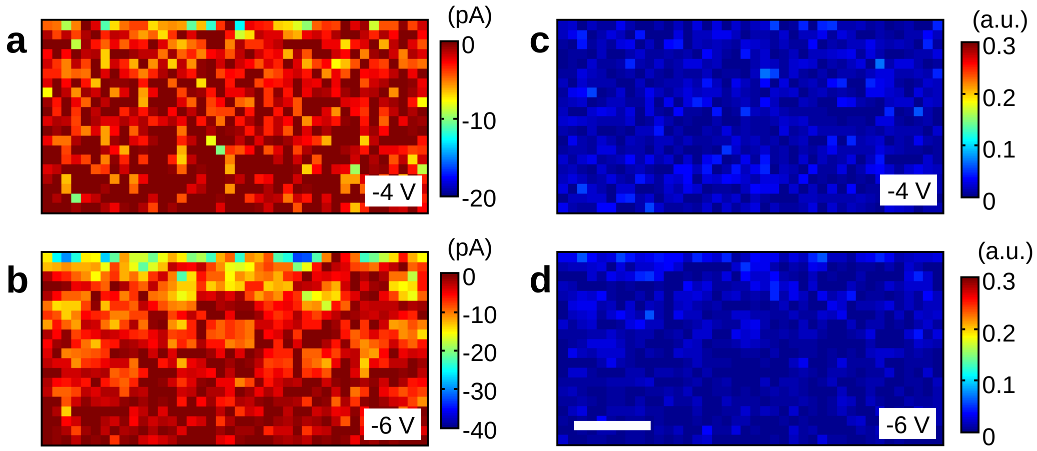

Supplementary Figure 10: Spatial maps of current response and relative FORC-IV loop area at different peak biases. Spatial maps of (**a**,**b**) current responses and (**c**,**d**) FORC-IV loop area at (**a**,**c**) -4 V and (**b**,**d**) -6 V. The image size is 250 × 125 nm^2. Scale bar, 50 nm.

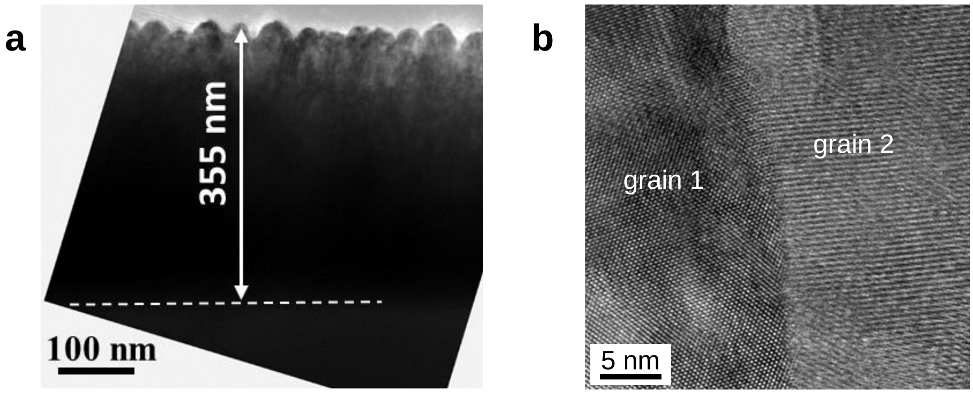

Supplementary Figure 11: Cross-sectional TEM images of plain SDC film. (**a**) Cross-sectional-view of pure SDC film. (**b**) High-resolution TEM image of the pure SDC film, exhibiting the existence of grains (1 and 2) and grain boundaries.

Supplementary Note 1: The strain state and oxygen sublattice at the SDC/STO interface. To get further insight into the conduction of oxygen ions at the STO/SDC interface, we have imaged the interface using atomic-resolution STEM HAADF imaging. As can be seen clearly in Supplementary Fig. 2, the sub-lattices of STO and SDC are compatible periodically (the white arrows). Namely, every seven (002) planes of STO fit almost perfectly with five (002) planes of SDC, indicative of coherent (or commensurate) interfaces. This coherent interface is achieved by the lattice matching equality of 7 × $d(002)_{STO}$ (13.66 Å) ≈ 5 × $d(002)_{SDC}$ (13.58 Å), where $d(002)_{STO}$ and $d(002)_{SDC}$ is the (002) lattice plane spacing for STO and SDC, respectively. Based on the STEM image observation, the unit cell constant of SDC is estimated to be about 5.467 Å, indicating a 0.6% tensile-strained (i.e., [(5.467 – 5.433)/5.433] × 100 ≈ 0.62%) in the SDC lattice. This strained interfacial region is on order of only 1 – 2 nm at the interface.

As demonstrated in the SPM data, the highest ionic conducting paths along the [001] direction were observed at the cores of SDC pillars rather than the SDC/STO interfaces in our nanoscaffold films. The only 0.6% tensile strain is not a large enough interfacial strain to induce the observed enhancement of ionic conductivity in the nanoscaffold SDC-STO films. For example, in the STO/YSZ/STO trilayers systems, the YSZ layers were tensile-strained by a large value of 7% to match the STO lattice[1]. Such enormous interfacial strain completely changes the O sublattice, leading to increased conductivity[1-3]. In our nanoscaffold film, considering the fact that the positions of the cations (e.g., Ce) near the interface vary gradually along the vertical direction, the O sublattice in our film would be also disordered slightly. However, this is a very different case to the STO/YSZ/STO trilayers, where there is a highly *disordered* O sublattice due to huge interfacial strain. In conclusion, the SDC/STO interface effect (i.e., the interfacial strain and the resultant O sublattice disorder) is not

significant, and thus, could not be the primary origin of ionic conductivity enhancement in our system.

Supplementary Note 2: Frequency-dependence of the real part of ac conductivity in a Pt/Nb:$SrTiO_3$ system. To check the formation of the Schottky barrier at the interface and its effect on the frequency (f)-dependence of the real part of ac conductivity (σ_{ac}'), we fabricated a simple Schottky junction with semiconducting Nb-doped $SrTiO_3$ (Nb:$SrTiO_3$) and high work-function metal Pt. As shown in Supplementary Fig. 4, this device exhibits forward rectifying current−voltage curves, showing clearly the formation of the Schottky barrier at Pt/Nb:$SrTiO_3$ interface. The inset in Supplementary Fig. 4 shows the f-dependence of σ_{ac}'. The value of σ_{ac}' is reduced with a decrease in f at high frequency and shows the plateau to be at intermediate frequency. However, it does not show a conductivity drop at low frequency as the nanocomposite films do (e.g., Please see the σ_{ac}' values at 450 K of Supplementary Fig. 4 and those at 443 K of Fig. 2a in the main text). Hence, it can be deduced that the electronic conduction occurs at low frequency in this Pt/Nb:$SrTiO_3$ device, and thus the Pt electrode does not block the charge carriers (i.e., holes or electrons). In conclusion, the electrode polarization effect (the conductivity drop below the plateau region at low frequency) is due to use of an ion-blocking electrode, as observed in Fig. 2a of the manuscript. This result is further evidence of the ionic nature of conduction in our nanoscaffold SDC-STO films.

Supplementary Note 3: Comparison of dc conductivity measured by dc and ac techniques. To check for possible electronic contribution to the ac conductivity measurements of the samples, we have measured dc conductivity using a dc technique, which is similar to the method carried out by García-Barriocanal *et al.*[1]. The measurements have

been done by applying a short pulse of a constant voltage using a computer-controlled Keithley 2440 source-meter. To better compare the dc conductivity results obtained by ac and dc techniques, the same electrode configuration was used in both measurements. As described in the manuscript, high-quality 150 nm-thick Pt top electrodes[4,5] deposited by dc magnetron sputtering were used as “oxygen ion blocking electrodes”. Temperature-dependent measurements were performed at a probe station equipped with a hot plate from 20 to 550 °C.

As can be seen clearly in Supplementary Fig. 5, for both films the dc conductivity measured by the dc technique (hereinafter, referred to as σ_{dc}[dc]) is lower than the values obtained from ac measurements (referred to as σ_{dc}[ac]) in the whole temperature measurement range. For the SDC-STO nanoscaffold film, σ_{dc}[ac] is 15 to 200 times higher than σ_{dc}[dc]. (Except for the two data points measured at 360 K and 402 K, σ_{dc}[ac] is at least two orders of magnitude higher than σ_{dc}[dc].) On the other hand, for the plain SDC film, σ_{dc}[ac] is 4 to 20 times higher than σ_{dc}[dc]. Considering the experimental geometry, the case of the plain SDC film can be regarded as a series circuit composed of Pt top electrode, SDC film and Nb:STO bottom electrode. Thus, the possible origin of σ_{dc}[dc] measured in the plain SDC film is only electronic conduction through the SDC layer because its electronic resistance should be much higher than other two electrodes. Similarly, for the case of the SDC-STO nanoscaffold film, σ_{dc}[dc] should come from electronic conduction though both the SDC nanopillars and the STO matrix. (The values of σ_{dc}[dc] in the SDC-STO nanoscaffold film is slightly higher than that in the plain SDC film. This can be explained by a parallel connection of multiple resistances, i.e., $1/R_{tot} = 1/R_{SDC} + 1/R_{STO} + 1/R_{interface}$, where R is the resistance.) Therefore, the fact that σ_{dc}[ac] $\gg$ σ_{dc}[dc] indicates that the electronic contribution to the ac measurements can be considered to be negligible, and thus, the measured ac transport definitely originates from the conduction of oxygen ions.

Supplementary References

1. Garcia-Barriocanal, J. *et al.* Colossal ionic conductivity at interfaces of epitaxial ZrO_2:Y_2O_3/$SrTiO_3$ heterostructures. *Science* **321**, 676-680 (2008).

2. Pennycook, T. J. *et al.* Seeing oxygen disorder in YSZ/$SrTiO_3$ colossal ionic conductor heterostructures using EELS. *Eur. Phys. J. Appl. Phys.* **54**, 33507 (2011).

3. Pennycook, T. J. *et al.* Origin of colossal ionic conductivity in oxide multilayers: interface induced sublattice disorder. *Phys. Rev. Lett.* **104**, 115901 (2010).

4. Gellings, P. J. & Bouwmeester, H. J. *Handbook of Solid State Electrochemistry*. (CRC Press, 2010).

5. Guo, X. & Maier, J. Ionically Conducting two-dimensional heterostructures. *Adv. Mater.* **21**, 2619-2631 (2009).
